# 2-Amino-3-Chlorobenzoic Acid from *Streptomyces coelicolor*: A Cancer Antagonist Targeting PI3K/AKT Markers via miRNA Modulation

**DOI:** 10.3390/ph18050620

**Published:** 2025-04-24

**Authors:** Ashraf Khalifa, Joseph D. Balthazar, Pandurangan Subash-Babu, Mohamed Y. Zaky, Zeinab A. El-Moaty, Hairul Islam M. Ibrahim

**Affiliations:** 1 Biological Science Department, College of Science, King Faisal University, P.O. Box 400, Al-Ahsa 31982, Saudi Arabia; 2 Division of Microbiology and Biotechnology, Entomology Research Institute, Loyola College, Chennai 600034, Tamil Nadu, India; 3 Department of Food Science and Nutrition, College of Food and Agricultural Sciences, King Saud University, P.O. Box 2455, Riyadh 11451, Saudi Arabia; 4 Molecular Physiology Division, Zoology Department, Faculty of Science, Beni-Suef University, Beni-Suef P.O. Box 62521, Egypt

**Keywords:** *Streptomyces*, column chromatography, bioautography, apoptosis, PCNA

## Abstract

**Background/Objectives:** Actinomycetes, particularly species within the *Streptomyces* genus, are renowned for their ability to produce a wide array of bioactive molecules with therapeutic potential. This study aimed to comprehensively investigate the antimicrobial and anticancer properties of *Streptomyces coelicolor* ERI-15, with a particular focus on a purified compound, 2-amino-3-chlorobenzoic acid (2A3CB), and its efficacy against microbial pathogens and breast cancer cell lines. **Methods:** Antimicrobial compounds were produced through fermentation techniques and isolated via column chromatography. Bioassay-guided fractionation was conducted against *Staphylococcus aureus* (ATCC 25923), methicillin-resistant *Staphylococcus aureus* (MRSA), *Escherichia coli* (ATCC 25922), and *Bacillus subtilis* (ATCC 441). Major fractions were further purified using preparative thin-layer chromatography (TLC). The structures of active compounds were elucidated using spectral analyses including IR, mass spectrometry, and ^1^H/^13^C NMR. The compound 2A3CB (*m*/*z* 171) was tested against MDA-MB-231 and 3T3 cell lines. Cytotoxicity was assessed by the MTT assay, and apoptotic mechanisms were explored via cell proliferation assays, dual fluorescent staining, migration and invasion assays, and analysis of apoptotic markers at mRNA and protein levels. **Results:** 2A3CB exhibited strong cytotoxic effects on MDA-MB-231 cells, with IC_50_ values of 26 µM, 5 µM, and 7.2 µM at 24, 48, and 72 h, respectively. It significantly inhibited cell proliferation and migration, and induced apoptosis via caspase-mediated pathways. Expression levels of PTEN, PCNA, BAX, and STAT3 were downregulated, suggesting inhibition of metastasis through the suppression of invasion and migration. **Conclusions:** The results demonstrate that 2A3CB, derived from *S. coelicolor* ERI-15, possesses potent antimicrobial and anticancer properties. Its ability to inhibit growth and induce apoptosis in MDA-MB-231 breast cancer cells highlights its potential as a natural therapeutic candidate for targeted cancer treatment, particularly in breast cancer progression.

## 1. Introduction

Soil microbiota is rich in bacterial communities, many of which remain poorly understood [1]. Among these microorganisms, *Streptomyces* stands out as a prominent genus of actinobacteria. These bacteria are particularly abundant in soil and are renowned for their ability to produce a wide variety of secondary metabolites that exhibit significant biological activities [2]. These bioactive compounds have garnered considerable attention for their potential applications as anticancer agents [3]. Notably, *Streptomyces* produces over 75% of all known antibiotics and yields other medically relevant compounds like antifungals, immunosuppressants, and anticancer agents [4]. The current understanding of the anticancer properties of soil-dwelling *Streptomyces* highlight the potential of this underexplored microbial resource for discovering new therapeutics to combat the growing burden of cancer, which poses a significant challenge to public health worldwide [2].

According to the most recent data from the American Cancer Society [5], the projected global cancer incidence rate is estimated to be around 20 million new cancer cases every year, with an estimated 9.7 million cancer deaths occurring worldwide, making cancer a leading cause of mortality globally. The urgency of cancer research has intensified due to the alarming increase in cancer incidence globally. While chemotherapy remains one of the most widely used treatments, its associated side effects have prompted researchers to explore naturally occurring compounds that may offer safer alternatives [6,7]. Natural products, particularly those derived from actinobacteria, are major sources of bioactive compounds [8]. The main goal of this study was to obtain a potent strain of *Streptomyces* from soil samples. The strain was characterized in terms of its cultural, micro morphological and biochemical parameters. In vitro methods were used to examine its ability to produce anti-cancer compounds. Several researchers were able to find a strain of *Streptomyces* that is morphologically different and isolated from northwestern mountain range of India. The isolated strain consistently showed higher anticancer activity against NCI-H460 and HeLa cells [8]. This activity is likely due to the production of bioactive metabolites, as the cell-free culture filtrates demonstrated pronounced effects. Additionally, the isolated actinobacteria produced other bioactive secondary metabolites, including amylase, protease, bacitracin, and catalase [9].

One of the largest groups of bioactive substance producing actinobacteria, particularly *Streptomyces* strains, have been extensively processed in this research. Actinobacteria are distinguished by a high guanine and cytosine content in their DNA, and they differ from other filamentous bacteria by containing mycolic acids long chain fatty acids that contribute to their unique properties [10,11]. Bioactive compounds, which include proteins, peptides, lipids, and fatty acids, have the capacity to inhibit the growth of various pathogenic bacteria and fungi. Actinomycete-derived bioactive compounds, particularly those from *Streptomyces thinghirensis*, can target pathogens like *Bacillus* spp. and *Escherichia coli,* as well as fungal pathogens and nematodes. They also enhance intestinal immune functions and offer protection against various pathogens [12]. Despite the promising potential of bioactive compounds, the cellular mechanisms through which they confer health benefits remain largely unexplored. The structural complexity of many bioactive compounds hinders our understanding of their specific mechanisms of action on cellular processes. To unlock the therapeutic potential of these compounds and determine their effective use in clinical settings, further studies are essential.

Natural products isolated from microorganisms have been the source of the most bioactive molecules available on the market today. Many natural compounds exert their anticancer effects through the induction of apoptosis, inhibition of cell proliferation, and suppression of metastasis [13]. For example, certain natural products act by disrupting the cell cycle and inducing oxidative stress, which leads to DNA damage and apoptosis [13]. Others, like flavonoids and alkaloids, are known to modulate signaling pathways, such as the PI3K/AKT, MAPK, and NFkB pathways that are critical for tumor growth, survival, and metastasis [14]. The broad range of bioactive natural products derived from actinomycetes, such as *Streptomyces*, have been shown to possess similar mechanisms, making them promising candidates for anticancer therapy [15]. Thus, the discovery of numerous antimicrobials and anti-proliferative agents from microbes, particularly actinomycetes and fungi, underscores their importance in modern medicine. The terms antibiotics and antimicrobial products are closely related, with antibiotics originally defined as substances produced by living organisms to kill or inhibit the growth of bacteria [16,17]. Although the term “antibiotic” now includes synthetic compounds, most marketed antibiotics are still based on natural chemotypes. For instance, 70 out of 90 antibiotics marketed in 2020 originated from natural sources [17]. The rise of antibiotic resistance is becoming a critical global issue, and alternative strategies for developing new antibiotics are gaining traction. One approach involves combining existing antibiotics with compounds that can reverse bacterial resistance. Another strategy focusses on developing inhibitors of bacterial efflux pumps, which contribute to resistance against various classes of antibiotics [18,19,20]. Generally, actinomycetes have been the most prolific group in antibiotic production, yielding numerous marketed antibiotics [21]. Despite extensive research, evidence suggests that only a small fraction of the species or genetically distinct strains of actinomycetes and fungi have been cultured [4,22,23].

The research gap in this study lies in the limited exploration of soil-derived *Streptomyces* strains for novel anticancer compounds. While *Streptomyces* is well known for producing bioactive metabolites, only a small fraction of its species have been thoroughly investigated for their therapeutic potential. Additionally, the specific mechanisms underlying the anticancer effects of many of these natural compounds remain largely unexplored. Given the potential significance of actinomycetes in various fields, this study was conducted to identify active molecules from *Streptomyces*, assess the anticancer properties of their bioactive compounds, and highlight their potential as novel therapeutics in the fight against cancer.

## 2. Results

### 2.1. Extraction, Purification, and Bioassay-Guided Isolation of Active Compounds

The separation and fractionation of compounds from *S. coelicolor* ERI-15 was performed using various solvent systems, leading to the identification of significant bioactive fractions. The cell-free supernatant was sequentially extracted with hexane, ethyl acetate, and chloroform. Among these, the crude ethyl acetate extract exhibited strong antimicrobial activity against all tested pathogens, with notable efficacy against *Staphylococcus aureus*.

**Figure 1:**
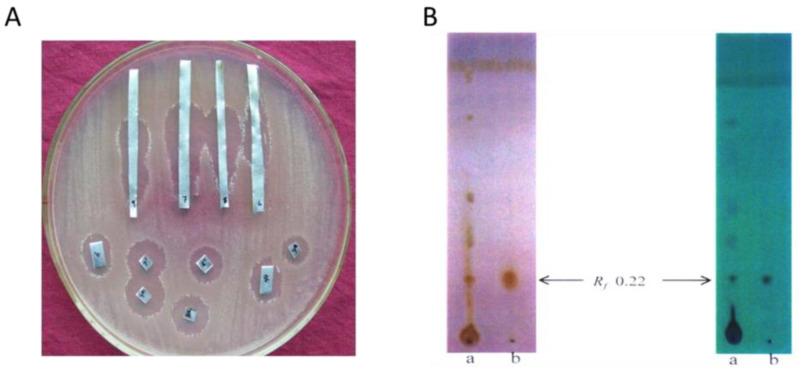
Bioautography and Thin Layer Chromatography (TLC) of Streptomyces coelicolor ERI-15 metabolites against methicillin-resistant staphylococcus aureus (MRSA). (**A**) Bioautography of major active fractions (7, 8, and 9) against methicillin-resistant *Staphylococcus aureus* (MRSA). This image displays an agar plate that has been uniformly inoculated with MRSA. Labeled paper strips (7, 8, and 9), each containing different fractions of metabolites extracted from *S. coelicolor* ERI-15, are placed on the surface of the agar. The presence of clear, circular areas surrounding these paper strips, known as zones of inhibition, indicates that the metabolites within fractions 7, 8, and 9 exhibit antimicrobial activity, effectively inhibiting the growth of MRSA. Control discs, labeled with numbers other than 7, 8, and 9, demonstrate either no inhibition or only minimal inhibition of bacterial growth. (**B**). TLC profile of Compound-3 of *S. coelicolor* ERI-15 a: Crude metabolites; b: Purified compound (solvent system: 4:6 Hexane-Ethyl acetate).

To further purify the bioactive compounds, the crude extract was subjected to silica gel (100–200 mesh) column chromatography and eluted stepwise with hexane and ethyl acetate, yielding 650 fractions (50 mL each). Based on TLC band profiles, these fractions were pooled into 12 sub-fractions, which were subsequently screened for antimicrobial activity. Fractions 7, 8, and 9 displayed significant antimicrobial effects and were further purified using preparative TLC, resulting in the isolation of three distinct compounds (Figure 1).

In the hexane:ethyl acetate solvent system, notable bioactivity was observed in multiple fractions, including Fraction 1 (TLC sub-fractions 1–50), Fraction 2 (77–89), Fraction 3 (139–152), Fraction 4 (174–195), Fraction 5 (249–294), Fraction 6 (295–305), Fraction 7 (306–348), and Fraction 8 (349–444). Additionally, the acetone:ethyl acetate system produced active Fractions 9 (486–516) and 10 (593–612), both of which demonstrated bioactivity. The methanol system yielded Fraction 12 (633–650), which was also identified as active (Table 1).

The antimicrobial activity of various fractions against selected bacteria, including *Staphylococcus aureus*, Methicillin-resistant *Staphylococcus aureus* (MRSA), *Bacillus subtilis*, and *Escherichia coli*, was evaluated based on inhibition zone diameters (Table 2).

The antimicrobial activity of various fractions against *Staphylococcus aureus*, MRSA, *Bacillus subtilis*, and *Escherichia coli* was assessed by inhibition zone diameters (Table 2). Notably, MRSA showed susceptibility to several fractions, especially Fr-5 (7 mm for *S. aureus*, MRSA, *B. subtilis*, and *E. coli*), along with Fr-6, Fr-7, and Fr-8. These results highlight the potential of these extracts in combating resistant infections, warranting further investigation into their active compounds for therapeutic development. Overall, the findings support the exploration of n

### 2.2. Characterization of the Compound-3 of S. coelicolor ERI-15

#### 2.2.1. Physical Properties Using TLC

The extracted compound was a pale-yellow fine crystal with UV-reactive properties and an Rf value of 0.22 in a 4:6 hexane:ethyl acetate TLC system. It was soluble in methanol, DMSO, and ethyl acetate but poorly soluble in water. The purified compound had a melting point of 193–194 °C, indicating its stability and purity.

#### 2.2.2. UV-Vis Spectrum

**Figure 2:**
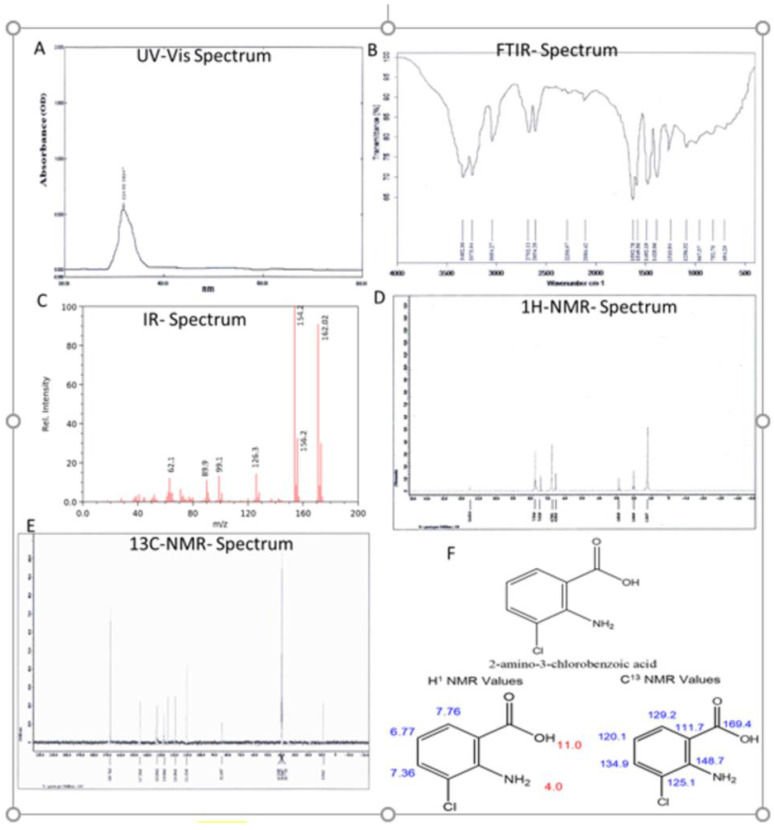
Chemical characterization of the compound-3 (2A2CB) of *S. coelicolor*. ERI-15. (**A**). UV Vis spectral data of pooled fraction-7–9, (**B**,**C**). FTIR spectral data analysis of Fractions 7–9. (**D**,**E**). ^1^H NMR spectrum of Compound-3 of *Streptomyces* sp. ERI-15. (**F**). Structural formula and name of the active molecule formula C_7_H_2_ClN_02_. Derived structure details from ^1^H and ^13^C NMR values. Its IUPAC name is 2-amino-3-chlorobenzoic acid (2A3CB).

The UV absorption spectrum of the purified compound in methanol (Figure 2) displayed a maximum absorption peak at 324 nm. No absorption was observed in the visible region.

The physiochemical structural analysis is illustrated in Figure 2. Figure 2A presents the UV-Vis spectrum of Compound-3 from *Streptomyces* sp. ERI-15. UV-Vis spectroscopy, a powerful analytical technique, identifies electronic transitions, providing insights into molecular structure and functional groups. The spectrum reveals distinct absorption peaks, indicating specific chromophores. The observed wavelengths suggest possible π-π* or n-π* transitions, reflecting the compound’s electronic properties.

#### 2.2.3. IR and Mass Spectrum

Figure 2B presents the infrared (IR) spectrum of Compound-3 from *S. coelicolor* ERI-15. The IR spectrum reveals key functional groups through characteristic absorption bands. Peaks at Vmax 3482 cm^−1^ correspond to hydroxyl or NH stretching, while Vmax 1594 cm^−1^ and Vmax 1554 cm^−1^ represent N–H stretching and bending, respectively. A peak at Vmax 1492 cm^−1^ suggests aliphatic CH_2_ stretching, and Vmax 1256 cm^−1^ is associated with C–O stretching. Additionally, Vmax 752 cm^−1^ corresponds to C–Cl stretching. The spectrum shows a molecular ion peak at *m*/*z* 300, indicating the molecular weight of Compound-3, with fragmentation peaks at *m*/*z* 285, 257, and 230 providing structural insights. The GC-MS spectrum further supports the molecular weight determination with a prominent peak at *m*/*z* 171.56 g/mol, confirming the molecular formula and aiding in the compound’s identification and characterization. IR peaks revealed that Aromatic Ring: C–H stretch (aromatic): around 3000–3100 cm^−1^.C=C stretch (aromatic ring): around 1600–1450 cm^−1^. Amino Group (NH_2_): N–H stretch: around 3300–3500 cm^−1^ (two peaks). N–H bend: around 1500–1600 cm^−1^.Carboxylic Acid Group (COOH): O–H stretch (acidic): broad peak around 2500–3300 cm^−1^. C=O stretch: around 1700 cm^−1^. C–O stretch: around 1200–1300 cm^−1^. C–Cl Bond: C–Cl stretch: around 700–800 cm^−1^. in IR peaks conclude that the IR spectrum of 2-chloro-3-aminobenzoic acid will show a combination of peaks corresponding to the characteristic vibrations of the aromatic ring, amino group, carboxylic acid group, and the C–Cl bond, allowing for its identification and characterization.

#### 2.2.4. NMR Spectra

##### H NMR Spectrum

Figure 2 shows the ^1^H NMR spectrum of Compound-3 isolated from *Streptomyces* sp. ERI-15. This spectrum provides insights into the hydrogen environment, aiding in the determination of the compound’s molecular structure. The spectrum displays sharp peaks at 86.78 and 87.41 ppm, corresponding to C–H protons in the benzene ring. A singlet at 11.61 ppm is attributed to the carboxylic acid proton. Chemical shifts between 0.5 and 2.0 ppm indicate aliphatic protons, while signals between 6.0 and 8.0 ppm suggest aromatic protons.

##### C NMR Spectrum

The ^13^CNMR spectrum showed peaks at 119.6, 129.5, and 133.9 ppm assigned for methine in the benzene ring. Quaternary carbon resonates at 147.2 ppm and carboxyl carbon resonates at 169.7 ppm (Figure 2E). Figure 2E presents the ^13^C NMR spectrum of Compound-3 isolated from *S. coelicolor* ERI-15. The ^13^C NMR spectrum shows distinct peaks at 119.6, 129.5, and 133.9 ppm, which are assigned to methane carbons in the benzene ring. Additionally, a peak at 147.2 ppm corresponds to a quaternary carbon, while a carboxyl carbon resonates at 169.7 ppm. These signals, along with others, help confirm the structural characteristics of Compound-3, indicating the presence of aromatic and functional groups

#### 2.2.5. Structure Elucidation

The purified Compound-3, identified as 2-amino-3-chlorobenzoic acid from *Streptomyces* sp. ERI-15, exhibits several notable physico-chemical properties (Table 3). It appears as pale-yellow fine crystals with a melting point of 193–194 °C, indicating high purity. Spectroscopic analyses reveal distinct features: UV-Vis spectroscopy shows absorbance peaks at 361 nm, 270 nm, and 220 nm, suggesting conjugated systems, while infrared spectroscopy highlights key functional groups with peaks at 3482 cm^−1^ and 1594 cm^−1^. The compound is soluble in methanol, DMSO, and ethyl acetate, with an Rf value of 0.22 in a 4:3 hexane:ethyl acetate system. A molecular weight search in databases such as PubChem and NIST identified a similar compound with a molecular mass of 171.58 g/mol, confirming the identity of the purified compound.

NMR analysis further confirms its structure. The ^1^H NMR spectrum shows a chemical shift at 171.56 ppm, indicating a de shielded hydrogen environment due to intra-molecular interactions. The ^13^C NMR spectrum reveals shifts at Δ6.78, 7.41, and others, confirming the carbon framework. Based on these physicochemical properties, the compound was identified as 2-amino-3-chlorobenzoic acid (2A3CB), with a molecular formula of C_7_H_2_ClN_02_. Its IUPAC name is 2-amino-3-chlorobenzoic acid, with a calculated molecular weight of 171.58 g/mol and an exact mass of 171.008 g/mol. These properties provide a deeper understanding of the compound’s behavior and its potential applications in pharmaceuticals and biochemical research.

#### 2.2.6. Cell Viability and Cytotoxicity Test

The active isolate *S. coelicolor* ERI-15 and its identified active molecule, 2A3CB, were tested for cytotoxicity against cancer cells using both the trypan blue exclusion test and the MTT assay, showing comparable cell death values relative to untreated controls. The active fractions from *S. coelicolor* ERI-15 exhibited significant toxicity against lung cancer cells. Specifically, active fraction 7 caused 74.86% cell death, leaving 26% viable cells after 72 h of incubation at a concentration of 500 µg/well. This fraction also achieved 50% cell death at 100 µg/well within 24 h. The IC50 for both the crude extracts and the active fractions was determined to be 110 µg/mL (Table 4).

#### 2.2.7. ALP Activity for Cell Proliferation Assay

The effect of active cytotoxic concentrations of *S. coelicolor* ERI-15 crude extracts and the active molecule 2A3CB on alkaline phosphatase (ALP) activity in the MDA-MB-231 cell line was assessed after 24, 48, and 72 h of treatment. A significant decrease in ALP activity was observed at 100 µg/mL for the crude extracts and at 5, 10, 20, 50, and 100 µM/mL for 2A3CB after 24 h of treatment. Prolonged exposure led to an even greater reduction in ALP activity. Notably, the lowest ALP activity was recorded in cells treated with *S. coelicolor* ERI-15 crude and 2A3CB after both the 24-h and 48-h treatment periods. These results suggest that 2A3CB has a pronounced inhibitory effect on ALP activity, indicating its potential to impact cell proliferation.

#### 2.2.8. Lactate Dehydrogenase Activity for Necrotic Analysis

**Figure 3:**
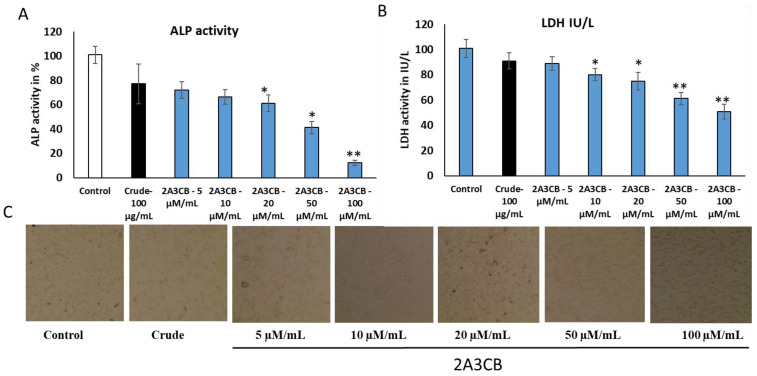
(**A**): Assay of alkaline phosphatase activity. This figure illustrates the alkaline phosphatase (ALP) activity in control and DAS-treated MDA-MB-231 cell lines. ALP levels are expressed as mU/mg protein, with values representing the mean of three independent experiments. (**B**): assay of Lactate Dehydrogenase activity. This figure presents the lactate dehydrogenase (LDH) activity in control and treated MDA-MB-231 cells. LDH units are expressed as µM of pyruvate liberated per minute per mg of protein. The values represent the mean of three independent experiments, converted to percentages of the control. Statistically significant differences compared to control cells are indicated by * *p* < 0.05, ** *p* < 0.01. (**C**) The results highlight the impact of 2A3CB treatment on ALP activity in both cell lines, demonstrating the potential effects of the tested compounds on enzyme activity.

The activity of lactate dehydrogenase (LDH) in control cells and those treated with *S. coelicolor* ERI-15 crude and 2A3CB is illustrated in Figure 3B. A notable decrease in LDH activity was observed in MDA-MB-231 cells treated with 2A3CB compared to control cells, indicating a reduction in necrotic activity. However, cells treated with 2A3CB showed increased LDH release, suggesting higher levels of cell damage or necrosis. These results help differentiate between cell shrinkage and lysis induced by different treatments. In conclusion, *S. coelicolor* ERI-15 crude and 2A3CB appear to induce apoptotic mechanisms of cell death, with the data highlighting differences in necrotic and apoptotic mechanisms of cell death associated with the treatments.

#### 2.2.9. Effect of Cell Migration

**Figure 4:**
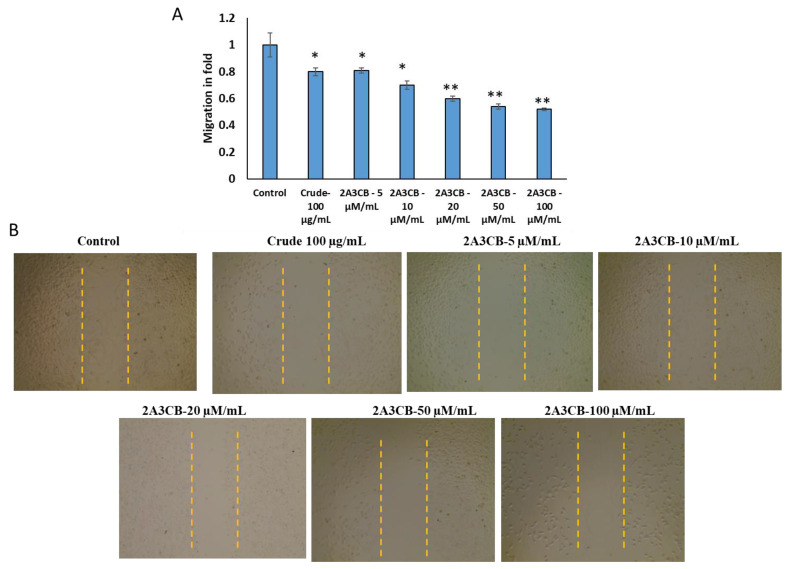
Scratch closures with tested *S. coelicolor* ERI-15 crude and 2A3CB treatment. (**A**). fold values was calculated using imageJ values between untreated and treated groups. (**B**). Scratch wound of remaining 5 upper images show *S. coelicolor* ERI-15 crude and 2A3CB treatment cells, monolayers were mechanically wounded with a 20–200 μL sterile pipette tip following treatment with *S. coelicolor* ERI-15 crude and 2A3CB treatment. Bar = 200 μm. Statistically significant differences compared to control cells are indicated by * *p* < 0.05 and ** *p* < 0.01 indicate statistically significant differences from control group.

In the wound healing assay, we examined cell migration in response to the mechanical scratch wound in the absence or presence of *S. coelicolor* ERI-15 crude and 2A3CB. Images of scratch areas from the time points 0 and 24 h are illustrated in Figure 4. Figure 4A shows the representative control at each time point indicating that the scratch was half closed within 24 h and completely closed in untreated control groups. Figure 4B represents the distance covered with and without treatment wells.

#### 2.2.10. Caspase Quantification for Activation of Apoptosis

**Figure 5:**
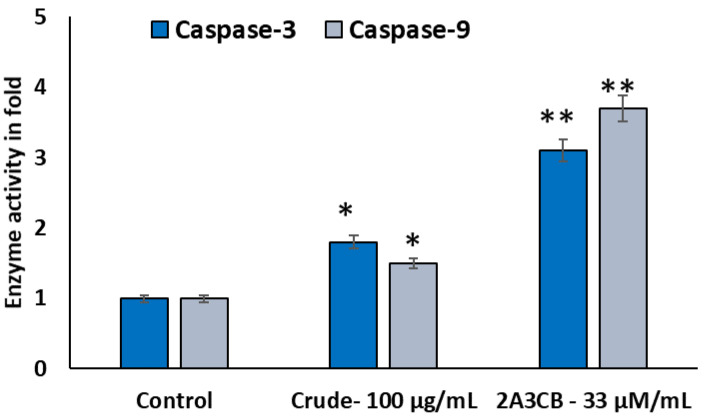
Enzymatic Caspase-9, and caspase-3 in MDA-Mb-231 treated with various concentrations of *S. coelicolor* ERI-15 crude and 2A3CB; C: control cells treated with DMSO 0.01% (vehicle). Data were shown as the mean ± SD from three independent experiments. One-way ANOVA followed by Tuckey’s multiple comparison tests were used to determine mean differences between groups. Statistical significance is shown in the figure as follows: by * *p* < 0.05 and ** *p* < 0.01 compared to control.

To confirm the apoptotic action of treatments, apoptotic cells were stained with acridine orange, which causes chromatin to accumulate and exhibit an orange-green coloration due to lysosomes around apoptotic bodies. As expected for basal levels, the control group showed Caspase-3 and Caspase-9 enzyme activities close to 1-fold. The crude extract (100 µg/mL) significantly enhanced the activity of both caspases (*p* < 0.05), suggesting a pro-apoptotic effect. However, 2A3CB (33 µM/mL) demonstrated a markedly stronger and highly significant increase in Caspase-3 and Caspase-9 activity (* *p* < 0.01), highlighting its potency as an inducer of apoptosis compared to the crude extract (Figure 5). 

#### 2.2.11. Effect of 2A3CB on Cellular DNA Damage Using Acridine Orange Staining for Apoptotic Evaluation

**Figure 6:**
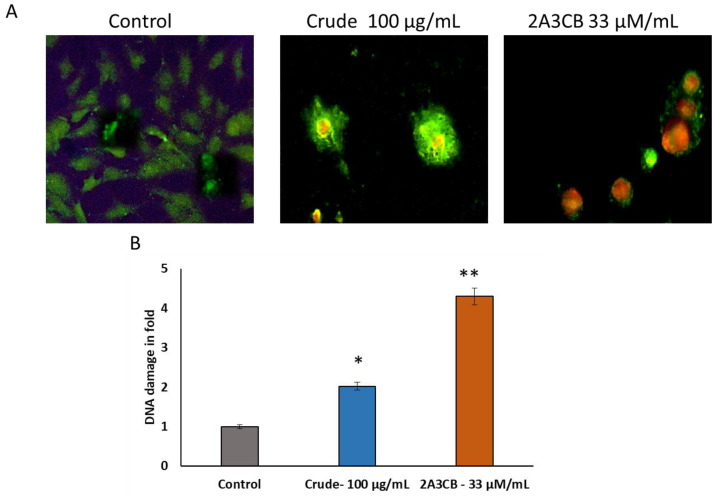
2A3CB activate the cellular nuclear damage in MDA-Mb-231 breast cancer cells lines. The DNA damage and formation linear strand was excited and showed yellow-orange fluorescence. Live and active cells emit green fluorescence, but apoptotic and necrotic cells emit total red-orange fluorescence evenly. (**A**). 2A3CB increased the double strand damage and apoptotic formation in ref orange colour. (**B**). DNA damage was expressed in yellow and orange colour quantification using imageJ software tool and valued revealed in fold change. Data are shown as mean ± SD from representative experiment studied in triplicate. * *p* < 0.05 and ** *p* < 0.01 compared to control., treated groups were compared with the DMSO group.

To confirm the apoptotic action of the treatments, apoptotic cells were stained with acridine orange, which highlights chromatin accumulation and results in an orange-green coloration due to lysosomes around apoptotic bodies. Figure 6B shows cells treated with *S. coelicolor* ERI-15 crude and 2A3CB, revealing the conversion of double-stranded DNA to single-stranded form. In contrast, Figure 6B shows chromatin clumping, fragmentation, and cell shrinkage after treatment. Treated cells exhibited chromatin clumping, fragmentation, shrinkage, and deformation, in contrast to control MDA-MB-231 cells (Figure 6A).

#### 2.2.12. Effect of 2A3CB on mRNA and miRNA Expression Level of PTEN, P13K, AKT Relevant miRNA miR-21, 23, 142 and 221

**Figure 7:**
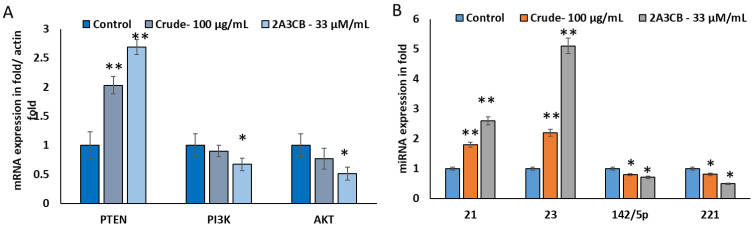
2A3CB abrogates tumorigenic properties. (**A**). 2A3CB treated cells breast cancer cells was examined for mRNA expression using real time PCR. The mRNA target of Pten, P13K, and AKT markers were quantified. (**B**). mRNA-specific miRNAs 21, 23, 142 and 221 were quantified using miRNA specific primers and real time PCR. Data are shown as mean ± SD from representative experiment studied in triplicate. Significant differences compared to the control group (DMSO) are denoted as follows: * *p* < 0.05 and ** *p* < 0.01.

As expected, the differences in genes expression were observed when cancer cells treated with *S. coelicolor* ERI-15 crude and 2A3CB on MDA-MB-231. According to Figure 7A, the PTEN gene was up-regulated while P13K and AKT were down-regulated in treated MDA-Mb-231 cells. However, there was no significant difference in P13K and AKT gene expressed in breast cancer MDA-MB-231 cells.

Figure 7B shows that *S. coelicolor* ERI-15 crude and 2A3CB significantly altered the expression of miR-21, miR-23, miR-142, and miR-221. MiR-142 and miR-221 targeted the PTEN gene, leading to its down regulation, while miR-21 and miR-23 targeted PIK3 and AKT, respectively. MiR-21 and miR-23 were up-regulated, while their target mRNA expressions were inhibited. These results confirm that *S. coelicolor* ERI-15 crude and 2A3CB reduce cancer-causing mRNA genes through reciprocal miRNA regulation.

## 3. Discussion

The bioactive compounds purified and identified from *S. coelicolor* ERI-15 crude included 2-amino-3-chlorobenzoic acid. This discovery is significant as it aligns with previous research highlighting the remarkable ability of actinomycete strains, particularly those within the *Streptomyces* genus, to produce a diverse array of antibiotics [24]. For example, *S. rochei* has been documented to produce potent macrolide antibiotics such as lankacidin, which has applications in treating bacterial infections [25], and borrelidin, another antibiotic known for its efficacy [26]. Additionally, this species is capable of synthesizing peptide antibiotics like streptothricin [27] and cis-2-amino-1-hydroxycyclobutane-1-acetic acid, which is recognized as a free amino acid herbicide, showcasing the versatility of *Streptomyces* in producing compounds with varied biological activities.

Similarly, *S. fradiae* has been reported to yield a range of antibiotics, including phosphoramide, tylosin, and neomycin [28], further emphasizing the potential of actinomycetes in pharmaceutical applications. The work of Lacey and Rutledge, [29] also supports this notion, as they documented four bioactive molecules from *Streptomyces* sp. TN97, which belong to three different chemical families: diketopiperazines, isocoumarins, and n-acetyltyramine. This diversity in antibiotic production not only highlights the rich biochemical potential of *Streptomyces* species but also opens avenues for discovering new antimicrobial agents that can combat the rising threat of antibiotic-resistant bacteria. The compounds identified in this study could lead to the development of novel therapeutic strategies and contribute to the ongoing search for effective treatments against various infectious diseases.

The first compound isolated from *S. coelicolor* ERI-15 crude and 2A3CB was dibutyl phthalate, which was identified through a comprehensive array of spectroscopic techniques, including UV-visible spectroscopy, IR, GC-MS, and NMR. This meticulous approach ensured a robust characterization of the compound. Other than this molecule, dibutyl phthalate has previously been recognized for its antimicrobial efficacy, particularly in the culture filtrate of *Streptomyces albidoflavus* 321.2, as reported by Roy et al. [30] (2006). Its active properties have also been documented in the context of Desulfovibrio desulfuricans [31], further illustrating its potential as a bioactive agent.

Dibutyl phthalate has also been isolated from various marine algae and has been utilized as a cathepsin B inhibitor, showcasing its versatility beyond antimicrobial applications [32].

A dark pink, thick amorphous compound was successfully purified from *S. coelicolor* ERI-15 crude and 2A3CB yielding an Rf value of 0.83 in a 7:3 hexane:ethyl acetate solvent system. Through careful spectroscopic analysis, including UV absorption measurements at 529 nm, as well as infrared (IR) and nuclear magnetic resonance (NMR) spectral analysis, this compound was identified as a hydroxyquinoline derivative. The antibacterial properties of hydroxyquinoline derivatives have been notably demonstrated, with the compound isolated from *S. coelicolor* ERI-15 crude and 2A3CB exhibiting significant antibacterial activity against *E. coli*.

The production of various pigments by actinomycetes, including hydroxyquinoline derivatives, is well documented in the literature. A prominent example is *Streptomyces* coelicolor, which is known for producing pigmented antibiotics such as actinorhodin and undecylprodigiosin, as noted by Chater and Hopwood [33]. These pigments not only contribute to the aesthetic qualities of the organisms but also play crucial roles in their ecological interactions and potential therapeutic applications. The findings regarding the hydroxyquinoline derivative further emphasize the rich biochemical diversity of *Streptomyces* species and their capacity to produce compounds with significant antibacterial properties, presenting promising avenues for the development of new antibiotics.

The third compound isolated from *Streptomyces* sp. ERI-15 was identified as 2-amino-3-chlorobenzoic acid, with the molecular formula C_7_H_2_ClN_02_. This compound was purified using a 4:6 hexane:ethyl acetate solvent system, yielding an Rf value of 0.22. Notably, 2-amino-3-chlorobenzoic acid exhibited potent antibacterial activity against methicillin-resistant *Staphylococcus aureus* (MRSA), highlighting its potential as a therapeutic agent in combating resistant bacterial infections.

Furthermore, compounds like 2-amino-3-chlorobenzoic acid, such as 3-chloroanthranilic acid (synonym for 2A3CB), is also produced by certain halophytic bacteria, have also exhibited notable bioactivity, as highlighted [34]. This connection emphasizes the importance of structural analogs in the development of bioactive compounds and their potential applications in medicine. The significant antibacterial properties of 2-amino-3-chlorobenzoic acid enhance our understanding of the biochemical profile of *Streptomyces* sp. ERI-15 and open avenues for future research to explore its mechanisms of action and possible clinical applications. This compound serves as an excellent example of the valuable contributions of microbial natural products to antibiotic discovery.

In addition to these findings, ALP plays a critical role in various biological processes, including metabolite transport across cell membranes, protein synthesis, secretory activities, and glycogen metabolism. ALP is also recognized as a tumor marker, making it useful for early cancer detection [35]. Changes in ALP levels can indicate alterations in membrane permeability and metabolic transport processes. These findings suggest that the compounds may influence cellular metabolic processes and membrane integrity, warranting further exploration of their implications in therapeutic contexts.

LDH is a vital cytoplasmic enzyme that facilitates the conversion of lactate to pyruvate and serves as an important marker for evaluating cell membrane integrity. Elevated LDH activity is frequently associated with malignant cells, reflecting increased membrane permeability and subsequent enzyme leakage. In our study, LDH levels exhibited stability across most treatment conditions, however, treatment with 2A3CB resulted in a notable 26% increase in LDH levels compared to control cells. This elevation indicates compromised cell membrane integrity and suggests the occurrence of necrosis.

In contrast, cells treated with 2A3CB maintained LDH levels like those of control cells, implying that while both treatments may lead to cell death, it was associated with necrotic cell death characterized by lysis mechanisms. These findings highlight the differential effects of the treatments on cell viability and membrane integrity, providing insights into the specific pathways through which these compounds exert their cytotoxic effects. This distinction is crucial for understanding the mechanisms of action of these treatments and their potential implications in therapeutic strategies targeting cancer and other conditions characterized by abnormal cell proliferation. Further investigation into the pathways involved could elucidate the broader implications of these compounds in the context of cellular health and disease. Additionally, while the findings of this study highlight the promising anticancer properties of 2A3CB, it is important to consider its limitations and potential side effects before assuming its clinical applicability. Furthermore, recent studies have demonstrated the promising potential of natural and nano-formulated compounds in cancer therapy. A synergistic anticancer effect of melittin and erlotinib in non-small cell lung cancer has been reported, highlighting the power of combinatorial approaches targeting key oncogenic pathways [36]. The biomedical efficacy of nanoparticle-based flavonoid delivery systems in treating various conditions including cancer, through improved bioavailability and targeted action, has also been emphasized [23]. Additionally, silver nanoparticles biosynthesized from *Bacillus* sp. KFU36 were shown to effectively induce apoptosis in breast cancer MCF-7 cells, underscoring the therapeutic potential of microbe-derived nanomaterials [37]. These findings support the relevance of natural bioactive agents, such as 2-amino-3-chlorobenzoic acid from *Streptomyces coelicolor*, in modulating cancer-related pathways like PI3K/AKT through miRNA regulation. Although 2A3CB exhibited significant cytotoxicity against MDA-MB-231 breast cancer cells and demonstrated effective modulation of apoptotic pathways, the compound’s selective toxicity, stability, and bioavailability in vivo need further investigation.

Additionally, while 2A3CB shows promising anticancer potential, its clinical applicability requires further evaluation. Key concerns include its selectivity, stability, and bioavailability in vivo. Long-term effects and possible off-target actions, especially on normal cells, need further investigation. Additionally, the compound’s pharmacokinetics, safety, and potential side effects, such as organ toxicity or immune reactions, should be assessed in pre-clinical models. While promising, 2A3CB’s therapeutic potential must be carefully weighed against these risks for safe clinical use.

## 4. Materials and Methods

Eagle’s Modified Minimum Essential Medium (EMEM, phenol red-free), and fetal bovine serum (FBS) were obtained from Sigma Chemical Co. (St. Louis, MO, USA) and an antibiotic solution containing 10,000 U/mL penicillin and 10 mg/mL streptomycin were sourced from Invitrogen (Carlsbad, CA, USA). These chemicals were stored in –20 °C in a cool, dry, place. The trypsin-EDTA mixture (0.25% trypsin and 0.02% EDTA) was acquired from Lonza Walkersville (Walkersville, MD, USA), These chemicals were stored in –20 °C in a cool, dry, place. p-Nitrophenyl phosphate was obtained from Sigma, USA, while dinitrophenylhydrazine was sourced from Merck, Mumbai, India, Store at +4 °C for short-term storage; for long-term storage, −20 °C is recommended. Caspase-specific substrates, 4-methyl-coumaryl-7-amide (MCA)-Leu-Glu-His-Asp-p-nitroanilide for caspase-9 and MCAAsp-Glu-Val-Asp-p-nitroanilide for caspase-3, were also from Merck, cDNA kit from takara, Japan and SYBR green master mix from Invitrogen, USA. Store at +4 °C for short-term storage; for long-term storage, −20 °C is recommended. All other reagents used in this study were obtained from standard suppliers and were of analytical grade or higher.

### 4.1. Fermentation

The fermentation was carried out in Modified Nutrient Glucose (MNG) medium using *S. coelicolor*. ERI-15 in a 3.0 L fermentor with a working volume of 2.0 L × 5 batches. The composition of the fermentation medium was as follows (g/L): peptone, 5; glucose, 20; sodium chloride, 3; calcium carbonate, 1.5; yeast extract, 3; and antifoam 204 (Sigma), 0.1. The pure starter culture of 1% (*v*/*v*) inoculum 2 × 10^6^ cfu/mL of *S. coelicolor*. ERI-15 was introduced into the fermentation medium using a peristaltic pump under aseptic conditions. Fermentation was carried out at 30 °C, with constant stirring at 350 rpm, filtered and sterile air was supplied using 0.2 micron Millipore fitters at an aeration rate of 1.0 vvm for 7 days. The sterility of the fermentation broths were continuously monitored for contaminants other than *S. coelicolor*. ERI-15. Following the incubation period, the culture broth was collected, and the cell-free supernatant was extracted sequentially with equal volume of Hexane, Ethyl Acetate, and Chloroform. The solvent phase was concentrated in a rotary vacuum evaporator to obtain the crude extract and concentrated. The resulting crude extract was then subjected to column chromatography to isolate the bioactive compounds.

### 4.2. Column Chromatography and Compound Isolation

The concentrated crude ethyl acetate extract of *S. coelicolor*. ERI-15 was loaded onto a silica gel chromatography column. The column was eluted using a stepwise increasing polarity of hexane and ethyl acetate in a ratio of 95:5. Collected fractions were pooled based on their TLC profiles. Subsequent antimicrobial activity of the sub-fractions was evaluated against test pathogens using the disc diffusion method following NCCLS standards. Major bioactive fractions were further tested against MRSA using the bio autography method. Fractions exhibiting antimicrobial activity were purified using preparative HPLC, leading to the isolation of bioactive compounds. The isolated bioactive compounds were structurally analyzed using GC-MS, IR, and both 1D and 2D NMR techniques.

### 4.3. Spectral Studies of the Isolated Bioactive Compounds

#### 4.3.1. UV-Visible Spectral Analysis

The UV-Visible spectral analysis of the purified antimicrobial compounds was conducted using a Shimadzu UV-2450 UV-Visible spectrophotometer (Shimadzu, Kyoto, Japan). The compounds were dissolved in methanol, and the spectrum was recorded over a range of 200–800 nm.

#### 4.3.2. Gas Chromatography-Mass Spectrometry Analysis (GC-MS)

The GC-MS analysis of the purified compounds were performed using a Shimadzu GC-MS-QP 2010 equipped with a DB-5 MS column (30 m × 0.25 mm i.d., 0.25 μm film thickness). The operating conditions were as follows: the column temperature was initially set at 50 °C for 1 min, then increased to 300 °C at a rate of 10 °C per minute, with a hold time of 10 min. Helium (99.9995% purity) was used as the carrier gas, flowing at a rate of 1.50 mL/min, with a split mode for sample injection. The injector temperature was maintained at 280 °C. The mass spectrometer operated with an ion source temperature of 200 °C and an interface temperature of 240 °C, scanning from 40 to 1000 Da.

#### 4.3.3. FT-IR Spectrum

The FT-IR spectra of the purified antimicrobial compounds were recorded using a Spectrum One model spectrophotometer (Perkin-Elmer Co., Waltham, MA, USA) with KBr pellets.

#### 4.3.4. Nuclear Magnetic Resonance Spectroscopy

Nuclear magnetic resonance spectroscopy for the purified compounds was conducted using ^1^H NMR and ^13^C NMR in CDCl_3_ or deuterated DMSO, with tetramethylsilane (TMS) as the internal standard, adjusting the sample concentration to 0.5% by weight for compounds 1, 2, and 3. High-resolution 1H NMR spectra were recorded on a Jeol ECA500 MHz spectrophotometer equipped with the δ version Iris platform, operating within a chemical shift range of 15 ppm to −5 ppm, utilizing 16,384 points, a pulse width of 6.575 µs, a relaxation delay of 5 s, an acquisition time of 1.308 s, and a field strength of 11.747 T. Similarly, high-resolution ^13^C NMR spectra were obtained under the same conditions, but with a chemical shift range of 225 ppm to −25 ppm, using 32,768 points, a pulse width of 3.4 µs, a relaxation delay of 2 s, an acquisition time of 0.83361 s, and maintaining the same field strength of 11.747 T.

### 4.4. Cell Culture

The human breast cancer cell lines MDA-MB-231 and 3T3 fibroblast cell lines grown in DMEM media supplemented with L-glutamine, 10% fetal bovine serum (Gibco, Thermo Fisher Scientific, Waltham, MA, USA), and antibiotics (penicillin 100 units/mL; streptomycin 100 units/mL). Cells were maintained in 75 cm^2^ tissue culture flasks at 37 °C in a humidified atmosphere with 5% CO_2_. After reaching confluence, the cells were trypsinized and plated on 6-well and 96-well plates, incubating for 12 h for attachment. Treated cells were lysed using 0.1% Triton X-100, and the cell lysates were used for biochemical assays.

### 4.5. MTT Cytotoxicity Assay

Cell seeding: MDA-MB-231 and 3T3 cells are plated in 96-well plates at 2 × 10^3^ cells/well in culture medium. Test compounds (crude and 2A3CB) are dissolved in 5% DMSO and diluted in culture medium to desired concentrations. Cells are exposed to compounds for 3 days. 5% DMSO treated alone cells were considered as control cells. 20 µL of MTT stock (5 mg/mL) is added per well (final concentration ~0.83 mg/mL in 120 µL total volume), followed by 1-h incubation at 37 °C. Medium is replaced with 50 µL 5% DMSO to dissolve formazan crystals. Note: Alternative solubilization reagents like SDS-HCl or acidified isopropanol are also used in other protocols. Absorbance is read at 570 nm using a microplate reader, with viability expressed as a percentage of untreated controls.

### 4.6. Alkaline Phosphatase Assay

Alkaline phosphatase (ALP) activity was measured in control and treated cells as a marker of cell differentiation [36]. The assay followed the method of Moss [33,36]. Briefly, 2A3CB treated cells with different concentration from 5 µM, 10 µM, 20 µM, 50 µM, 100 µM cells were centrifuged at 4000× *g*, resuspended in PBS, and sonicated. 5% DMSO treated alone cells were considered as control cells. Supernatants are collected after debris removal via centrifugation and stored at −70 °C. pNPP substrate is added to lysates and incubated at 37 °C. Liberated p-nitrophenol is quantified at 405 nm using a spectrophotometer. (Shimadzu spectrophotometer). Express results as fold-changes relative to controls to account for plate-to-plate variability.

### 4.7. Lactate Dehydrogenase (LDH) Assay

LDH activity was measured following the method of [37]. Cancer cell lines were treated with 2A3CB with different concentration from 5 µM, 10 µM, 20 µM, 50 µM, 100 µM cells for 12 h incubation, attached cells were lysed using 0.1% Triton X-100 and subjected to two cycles of freezing and thawing. 5% DMSO treated alone cells were considered as control cells. Lysates are mixed with substrate buffer (0.5 mM lactic acid, 0.1 N NaOH, 0.1 M glycine buffer) and 0.02% dinitrophenylhydrazine. Absorbance is read at 460 nm. Shimadzu spectrophotometer. Express results as fold-changes relative to controls to account for plate-to-plate variability.

### 4.8. Cell Migration—Wound Healing Assay

MDA-MB-231 cell lines was seeded at a 5 × 10^4^ cell density in 24-well plates and then allowed to adhere overnight. At confluence, a wound was created across each well using the WoundMaker device. The cells were then treated with different concentrations of 2A3CB (5 µM, 10 µM, 20 µM, 50 µM, 100 µM) and incubated for 16 h. Then, pre-warmed medium or sample was added again, and pictures were taken. The scratch closure was monitored and imaged in 24-h intervals using a Leica 3000 microscope (Neu-Isenburg, Germany) at 4× magnification and 1/3700 s exposure time. The percentage of open wound area was plotted over the time for each concentration. Data are presented as mean ± SD. Three to six replicates were included in the analysis and an unpaired Student’s *t*-test was performed. Significance was considered at *p* < 0.05. The distance was calculated using standard microscopic image calculator (ImageJ software, version 1.53t, National Institutes of Health, Rockville Pike, MD, USA).

### 4.9. Quantification of Apoptosis by Caspase-9 and Caspase-3

Caspase-9 and caspase-3 activities in the treated cell extracts were measured colorimetrically based on the release of free p-nitroaniline (pNA) from the hydrolysis of specific chromogenic substrates (MCA-Leu-Glu-His-Asp-p-nitroanilide for caspase-9 and MCAAsp-Glu-Val-Asp-p-nitroanilide for caspase-3). The optical density of free pNA, proportional to caspase activity, was measured at 405 nm.

### 4.10. Acridine Orange Staining of Apoptotic Cells

Acridine orange (AO) staining was performed to differentiate between double-stranded (ds) and single-stranded (ss) nucleic acids. AO emits green fluorescence when binding to dsDNA and red fluorescence with ssDNA or RNA. A549 cells were cultivated overnight in a 24-well plate containing the test compounds. After washing and centrifugation, the cell pellet was resuspended in PBS, treated with RNase A, and incubated. Following a brief treatment with 0.1 N HCl, AO solution was added, and the cells were observed under fluorescent microscopy with a green filter.

### 4.11. Apoptotic Gene Expression Using Real-Time PCR

Gene expression analysis was conducted via real-time PCR. Total RNA was extracted from ACB-treated MDA-231 breast cancer cells using the High Pure RNA isolation kit (Roche Diagnostics, Indianapolis, IN, USA). RNA purity and integrity were assessed using a NanoDrop spectrophotometer. cDNA was synthesized using the cDNA Transcriptor First Strand cDNA synthesis kit. The cDNA mixture was incubated in a thermal cycler under specified conditions, and primers for target and reference genes were designed using primer design software (version 0.4.0).

### 4.12. Statistics

Results were expressed as mean ± standard deviation. Spectrophotometric measurements from the MTT assay, biochemical assays, and PCR results in MDA-231 cells were evaluated statistically using the student’s *t*-test via GraphPad Prism (version 5.0; GraphPad Software Inc., San Diego, CA, USA). A significance value of *p* < 0.05 was accepted

## 5. Conclusions

This study highlights the significant therapeutic potential of bioactive compounds derived from *Streptomyces* sp. ERI-15, especially in combating two of the most pressing global health threats: antimicrobial resistance and cancer. Notably, 2-amino-3-chlorobenzoic acid demonstrated strong antimicrobial activity, particularly against multidrug-resistant pathogens such as MRSA and *E. coli*, emphasizing its promise as a lead candidate in the development of next-generation antibiotics. Furthermore, the modulation of crucial cancer-related biomarkers—alkaline phosphatase and lactate dehydrogenase —indicates notable anticancer properties, suggesting a potential role in future cancer therapeutics. These findings not only reinforce the role of *Streptomyces*-derived metabolites as a prolific source of pharmacologically active molecules but also pave the way for dual-purpose therapeutics that address both microbial infections and malignancies. The dual functionality of these metabolites opens promising new avenues for research and drug development, further supporting the importance of microbial natural products in modern medicine.

## Figures and Tables

**Table 1:** Illustration of silica gel column chromatography and separation of compounds from the crude ethyl acetate extract of *Streptomyces* sp. ERI-15.

Fraction No.	Solvent System (%)		Fractions	TLC Based Sub Fractions	Bioassay Guided Significant Fractions
	Hexane:Ethyl Acetate				
1–13	35	65	51–305	1–6	
14	30	70	306–348	7	1
15	25	75	349–444	8	2
16	20	80	445–469		
17	10	90	470–475		
18	5	95	476–485		
Acetone: Ethyl Acetate					
19	10	90	486–516	9	3
20	25	75	517–552		
21	50	50	553–572		
22	75	25	573–592		
23	90	10	593–612	10 & 11	
24	100	0	613–632		
Methanol					
25	100		633–650	12	

**Table 2:** Bioassay-guided identification of active fractions against selected microbes. Fraction spot was made in aluminum coated silica plate with 10.

Sub Fractions	*S. aureus* ATCC 25923 (mm)	MRSA (mm)	*B. subtilis* MTCC 441 (mm)	*E. coli* ATCC 25922 (mm)
Fr-1–4	Nil	Nil	Nil	Nil
Fr-5	7	7	7	7
Fr-6	10	9	10	11
Fr-7	14	14	16	14
Fr-8	12	13	14	13
Fr-9	12	12	13	13
Fr-10	9	7	9	8
Fr-11	8	7	9	8
Fr-12	9	9	8	Nil

**Table 3:** Physico-chemical properties of the purified compound-3 from *Streptomyces* sp. ERI-15.

Physico-Chemical Parameter	Property
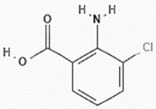	
Appearance	Pale yellow fine crystal
Melting point	193–194 °C
UV-Vis λMax methanol (nm)	361; 270; 220
IR (KBr) cm^−1^vmax	3482; 1594; 1554; 1492; 1256; 752
Solubility	Methanol; DMSO; Ethyl Acetate
Rƒ	0.22 (4:3 Hexane:Ethyl Acetate)
Mass spectrum (*m*/*z*)	171.56
^1^H NMR (Deuterated DMSO 500 MHz)	Δ6.78; 7.41; 11.61
^13^C NMR (Deuterated DMSO 100 MHz)	ppm 119.6; 129.5; 133.9; 147.2; 169.7

**Table 4:** Cell viability in MDA-MB-231 breast cancer cells.

Treatment	Concentration	Cell Viability in Percentage of Control Cells		
		24 h	48 h	72 h
crude	50 µg/well	92.2 ± 5.8	88.5 ± 3.71	86.4 ± 5.1
	100 µg/well	79.6 ± 3.3	70.45 ± 1.8	56.78 ± 6.1
	500 µg/well	64.21 ± 1.8	48.45 ± 3.73	41.8 ± 1.8
2A3CB	50 µg/well	62.45 ± 2.2	60.45 ± 5.8	66.78 ± 3.76
	100 µg/well	52.78 ± 3.71	36.48 ± 4.7	33.7 ± 4.1
	500 µg/well	41.2 ± 3.4	30.47 ± 1.8	26.12 ± 2.1

## Data Availability

Available from the corresponding author upon request.
